# Rasch analysis of the Persian version of PedsQL^TM^ Oral Health Scale: further psychometric evaluation on item validity including differential item functioning

**DOI:** 10.15171/hpp.2016.23

**Published:** 2016-08-10

**Authors:** Chung-Ying Lin, Santhosh Kumar, Amir H. Pakpour

**Affiliations:** ^1^The Hong Kong Polytechnic University, Hung Hom, Hong Kong; ^2^Griffith University, Gold Coast, Australia; ^3^Social Determinants of Health Research Center, Qazvin University of Medical Sciences, Shahid Bahounar BLV, Qazvin, Iran

**Keywords:** Children, Construct validity, Differential item functioning, Oral health, Quality of life, Rasch

## Abstract

**Background: ** The study aimed to further evaluate the psychometric properties of one recently developed oral health related quality of life (OHRQoL) instrument (PedsQL Oral Health Scale), including student self-report and parent-proxy report. Specifically, we tested the item validity,threshold order, local dependency, and differential item functioning (DIF) across gender and rater.

**Methods:** This is a cross-sectional study, and study population was recruited in Qazvin, Iran using one-stage sampling with the unit of school. Students and their parents (1529 dyads) separately completed the Persian version of PedsQL Oral Health Scale. The psychometric properties were analyzed using Rasch rating scale model, including item validity, threshold order for response categories, and DIF across gender (boys vs. girls in student self-report) and rater (student self report vs. parent-proxy report).

**Results:** All items had satisfactory in fit and outfit mean square error. One disordering category (the response of often) was found in parent-proxy report, while all categories were ordered in student self-report. All items were DIF-trivial across gender and rater.

**Conclusion: ** PedsQL Oral Health Scale is a valid instrument to measure OHRQoL. However, our results indicated that the parent-proxy report was inferior to the student self-report, and healthcare providers should primarily use the student self-report.

## Introduction

Over the last decades, the concept of quality of life (QoL) in relation to general and oral health has received increased attention,^1^ and many instruments have been developed because of this growing importance.^2^ However, the development of valid self-reported measures of oral health related QoL (OHRQoL) in children has occurred only in the last decade, prior to which parents were used as proxy.^3^ Using child self-report to accurately measure the QoL for children is a trend,^4,5^ and several child self-reported OHRQoL instruments have been proposed and found to be valid, viz., Child Oral Health-related Quality of Life,^6^ Child Oral Health Impact Profile,^7^ Child Oral Impacts on Daily Performances^8^ and Scale of Oral Health Outcomes for 5-year-old children.^9^

In addition to the above mentioned OHRQoL instrument, a feasible and efficient measure called Pediatric Quality of Life Inventory^TM^ (PedsQL) Oral Health Scale has been developed very recently in 2009. The PedsQL Oral Health Scale has been designed as a 5-item questionnaire including child self-reports and parent-proxy reports. In addition, the PedsQL Oral Health Scale can be used along with PedsQL 4.0 Generic Core Scales and disease-specific modules because oral health stands as a specific condition which is not measured by generic and disease-specific instruments.^10^ Furthermore, the PedsQL Oral Health Scale has been translated to Brazilian Portuguese^11^ and Persian^1^ versions with acceptable validity and reliability.

Although PedsQL Oral Health Scale has satisfactory psychometric properties among different language versions,^1,10,11^ we considered its evaluation is still underdeveloped. In order to well understand the properties of the QoL instruments, including the strengths and drawbacks, psychometric theories involved in developing the rating scales are suggested.^12^ Classical test theory (CTT) and item response theory (IRT) are two types of analytical strategies which can be used for this purpose.^13^ CTT emphasizes on the total summated scores in contrast to IRT which measures the properties of each individual item in reference to the data related to the latent trait.^12^ IRT allows analyzing scoring data by modeling both item and respondent characteristics concurrently and thus is advantageous over CTT.^14^ Therefore, IRT has become a method of choice and state of art in psychometric evaluation. There are three types of IRT model based on the estimated parameters, and the simplest model is the one-parameter logistic model, which is well-known as the Rasch model.^15^ Rasch analysis is based on assumptions that the scale is unidimensional and a person’s response to each item is independent of their response on other items.^12^

The Rasch model is based on the concept put forward by a Danish mathematician, Georg Rasch,^16^ and it involves testing the summated ordinal score obtained from multi-item instruments against the Rasch measurement model.^17^ The fit statistics obtained from Rasch analysis demonstrate the extent to which various items in the instrument describe the group and that how well the subjects fit the group.^16,17^ Rasch analysis also helps in evaluating the psychometric properties, such as item difficulty hierarchy and person separation statistics.^18^ The Rasch rating scale model has been widely used in psychometric analysis of various OHRQoL questionnaires^19,20^ and various modules of PedsQL.^12,17^ However, to the best of our knowledge, PedsQL Oral Health Scale has never been examined using Rasch analysis. In this study, therefore, we aimed to evaluate the validity of Persian PedsQL Oral Health Scale using Rasch analysis.

## Materials and Methods

The study was a cross-sectional study that conducted in Qazvin (a city near Tehran with a population of about 453554 inhabitants.) between May to September 2014. The socioeconomic status in Qazvin is comparable to the average of Iranian. Participants were secondary school students of ages 13-18 years. The students were approached through a list of high schools in the Qazvin city, and the potential candidates were public schools. A one-stage sampling procedure was used: The stage unit was school. Eight schools were randomly selected from 47 high schools in the Qazvin city. Letters detailed describing the project and informed consents were given to all students of the eight schools. Students with intellectual disability (as assessed by a trained psychiatrist) were excluded from the study. The students took the letters and consents home to inform their parents, and those who returned their written informed consents were included in the study (n=1529). The PedsQL Oral Health Scale was completed by the students in their classroom under supervision while the parents filled the forms at home.

### Measures

Demographic characteristics

A questionnaire was used to gather information on the socioeconomic characteristics, including age, gender, frequency of dental brushing and dental flossing, parents’ educational level and household income.

Oral health related quality of life 

The OHRQoL was assessed by the PedsQL Oral Health Scale.^10^ The PedsQL Oral Health Scale is a self-reported measure with five items (Appendix A). There are two parallel forms for the PedsQL Oral Health Scale: A child self-report form and a parent-proxy report form. Because our participants were all students, the child self-report in our study was defined as student self-report. All scores are rated on a 5-point Likert scale ranging from 0 (*never a problem*) to 4 (*almost always a problem*) and each item score is linearly transformed into a 0-100 scale. The average score of the 5 item can then be calculated to represent the total score of PedsQL oral health scale, and a higher score indicates a higher OHRQoL. The Persian version of the PedsQL Oral Health Scale was found to be highly valid and reliable for using in Iranian children and adolescents.^1^ For example, the test-retest reliability for 1 month is high (intraclass correlation coefficient=0.86 and 0.81), the internal consistency is excellent (α=0.79 and 0.89), construct validity is supported (comparative fit index=0.99; root mean square error of approximation=0.028 and 0.052), and known-group validity is good (children with decayed, missing and filled teeth have significantly lower score in PedsQL Oral Health Scale than those without decayed, missing and filled teeth do).

### Data analysis 

Sample size for Rasch analysis should be at least of 25 × numbers of categories in the response.^27^ Because we used 5-point Likert scale, the participants should be more than 25 × 5=125, and our participant number (n=1529) was sufficient. Before testing psychometric properties of each item, we reversely recorded the item scores of the PedsQL Oral Health Scale. That is, we used 0 to represent *almost always a problem*, and 4 to represent *never a problem*. All the following Rasch analyses used the recorded scores. We applied the Rasch rating scale model (RSM) to examine the PedsQL Oral Health Scale, respectively for the student self-report and the parent-proxy report, and used infit mean square error (MnSq) and outfit MnSq to test the unidimensionality of each item. The criteria of infit and outfit MnSq were set at 0.6 to 1.4,^12^ and MnSq> 1.4 suggests an out-of-concept item, while MnSq <0.6 a redundant item.^21^ Item difficulty with the unit of *logit* (a standardized score with mean as 0 and SD as 1) was also calculated for each item in both student self-report and parent-proxy report. In addition to testing unidimensionality, we also examined the separation reliability and indices, threshold order, local dependency, and differential item functioning (DIF) for the PedsQL Oral Health Scale.

The separation reliability include person separation reliability (measuring the reproducibility of the person ordering) and item separation reliability (detecting the reproducibility of the item difficulty); separation index include person separation index (discriminating the respondents into different clusters based on respondents’ ability) and item separation index (separating the items into different levels based on items’ difficulty).^22^ The acceptable values were 0.7 for reliability and 2 for index.^12,23^

The threshold order was examined using average measure, step measure, and fit statistics. Because we anticipated that the response should be located in their expected order (i.e., the response of 0 should be to easier than the response of 1, 1 easier than 2, and so on), both average and step measures should monotonically increase with the responses.^24^ In addition, some researchers^12,23^ suggest using the infit and outfit MnSq with the range of 0.6 and 1.4 to additionally examine the threshold order. The local dependency was evaluated using Pearson correlations (r) of the Rasch residuals between every two items, and an r=0 indicates perfect independent for the two items. However, the 0 relationship is practically unrealistic,^25^ and an alternative is using an absolute r ≤ 0.4.^21^

The DIF analysis was conducted using only student self-report for gender, and using both student self-report and parent-proxy report for rater. The DIF across gender examined that whether males and females interpret the items of PedsQL Oral Health Scale differently, and DIF across rater investigated that whether students and parents perceive a different OHRQoL for students. Ideally, the items should be DIF-free (i.e., a non-significant *t* test for two groups), while DIF-trivial (i.e., a DIF contrast < 0.5 *logits*, which means an odds ratio of 1.65) is also acceptable, for researchers comparing item scores across groups.^26^ In addition, the missing data did not impact the estimation in Rasch because the expected marginal scores are computed from non-missing observations, and missing data were skipped over in these additions.^27^ All Rash analyses were done using Winsteps.^27^

## Results

In total, 1529 students participated in the study, and their mean (SD) age was 15.05 (3.16) years. Most of the students were female (54.61%). The mean educational years in school for father and mother were 8.35 (5.43) and 6.59 (4.63) years, respectively. Approximately one fifth (n=321) of the students reported that they brushed their teeth twice a day. Only about one fifth (n=321) of students indicated that they brushed their teeth twice a day, and approximately half of the students reported that they never used dental floss (n=706). An average household monthly income was reported as US$ 246.62 with an SD of 178.88. Participant demographic characteristics are shown in Table 1.

The performance of the rating scale is shown in Table 2, and the most difficult item was OH2 (*Having tooth pain when eating or drinking something hot, cold, or sweet*; 0.78 and 0.47 *logits*) and the easiest item was OH4 (*Having gum pain*; −1.01 and −1.12 *logits*). In addition, except for the item OH5 on parent-proxy report (*Having blood on toothbrush after brushing teeth*) had a slight misfit based on infit MnSq (1.41), all other items fit well in the underlying construct of OHRQoL.

**Figure 1:**
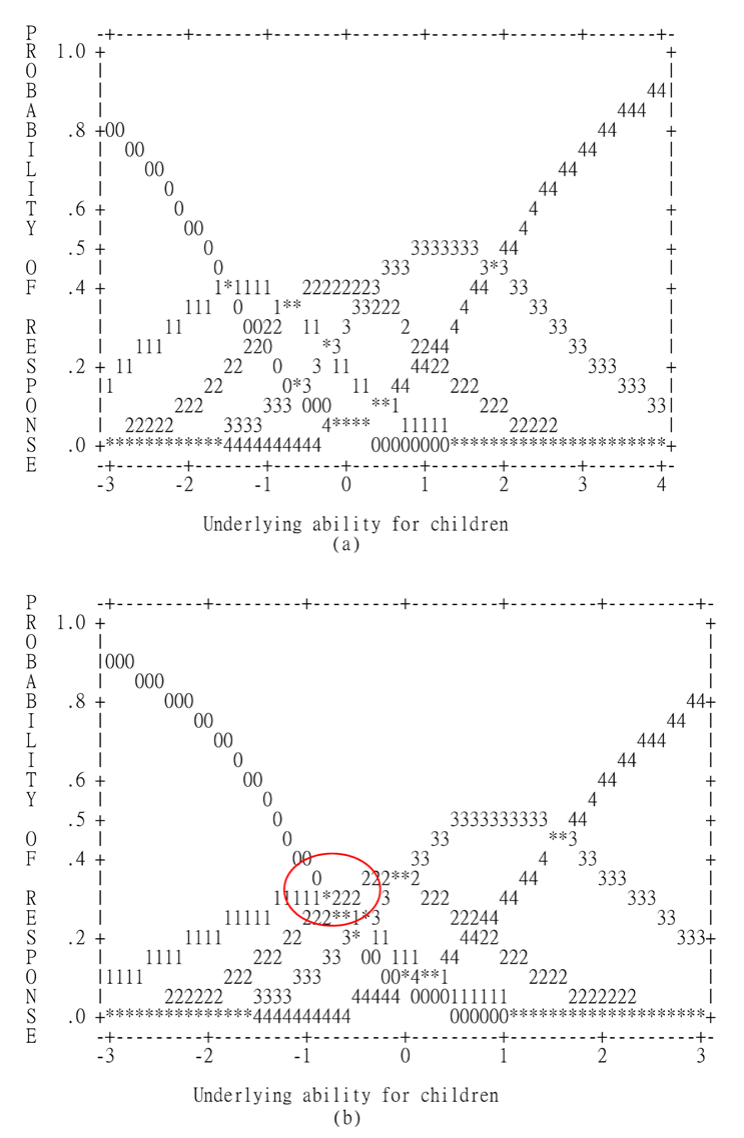
Probabilities of each response for PedsQL Oral Health Scale.
Legends: 0=almost always; 1=often; 2=sometimes; 3=almost never; 4=never. (a) is for student self-report and (b) for parent-proxy report.

The person separation reliability and separation index were slightly low for both the student self-report (reliability=0.63 and index=1.32) and the parent-proxy report (reliability=0.72 and index=1.59). However, the item separation reliability and separation index were excellent for both the student self-report (reliability=0.99 and index=12.80) and parent-proxy report (reliability=0.99 and index=13.72). The performance of threshold order is presented in Table 3, and the average measure was monotonically increased by responses (i.e., the smallest value in 0 and the largest value in 4). Though all infit and outfit MnSq fell in the reasonable range, a disordering category (i.e., the response of 1) was found for parent-proxy report based on the step measure. We further visualized the threshold disorder in Figure 1, and we could clearly see that parents tended not to rate the response of 1 (i.e., often) as indicated by the circle. That is, the probability of rating shifted from 0 to 2. The probability of response 1 was lower than that of response 0 when the underlying ability for children was less than -1; the probability of response 1 was lower than that of response 0 when the underlying ability was greater than -1.

No local dependency was found for student self-report (absolute r=0.14 to 0.38). However, two absolute *r* coefficients were higher than the recommendation though they were slightly higher (0.42 and 0.44; Table 4). In addition, all items were DIF-trivial across gender and rater though three items on parent-proxy report were not DIF-free across rater. Items OH2 (*Having tooth pain when eating or drinking something hot, cold, or sweet*; *P*<0.01) and OH4 (*Having gum pain*; *P*<0.01) were found to be more difficult for students than for parents; contrarily, item OH5 (*Having blood on toothbrush after brushing teeth*; *P*<0.01) was easier for students than for parents (Table 5).

**Table 1:** Demographic characteristics of the sample of Iranian children

	**n (%)**
Age (year)^a^	15.1 (3.2)
Gender	
Boys	694 (45.4)
Girls	835 (54.6)
Father's educational year^a^	8.4 (5.4)
Mother's educational year^a^	6.6 (4.6)
Tooth brushing	
Never	111 (7.3)
Less than once a month	78 (5.1)
Less than once a week	82 (5.4)
Once a week	216 (14.1)
Once a day	721 (47.2)
Twice a day	321 (21.0)
Dental floss	
Never	706 (46.2)
Less than once a month	169 (11.1)
Less than once a week	184 (12.1)
Once a week	225 (14.7)
Once a day	245 (16.0)
Monthly family income	
$0-500	489 (32.0)
$500-800	802 (52.4)
> $800	238 (15.6)

^a^Presenting as mean (SD).

**Table 2:** Item difficulty and fit statistics for PedsQL Oral Health Scale

**Scale and item**	**n**	**Mean (SD)**	**Difficulty**	**Infit**	**Outfit**
Child self-report	1529	80.46 (18.95)			
OH1: I have tooth pain	1525	79.48 (25.71)	0.17	0.89	0.89
OH2: I have tooth pain when eating or drinking something hot, cold, or sweet	1526	72.26 (28.00)	0.78	0.91	0.88
OH3: I have teeth that are dark in color	1523	80.73 (23.12)	0.05	0.78	0.81
OH4: I have gum pain	1522	89.17 (21.85)	-1.01	1.25	1.03
OH5: I have blood on toothbrush after brushing teeth	1527	81.04 (25.06)	0.01	1.35	1.33
Parent-proxy report	1496	74.99 (26.37)			
OH1: Having tooth pain	1525	74.26 (30.51)	0.15	0.86	0.86
OH2: Having tooth pain when eating or drinking something hot, cold, or sweet	1525	70.64 (31.36)	0.47	0.90	0.92
OH3: Having teeth that are dark in color	1525	75.77 (29.51)	0.02	0.82	0.89
OH4: Having gum pain	1520	85.02 (28.84)	-1.12	1.11	0.87
OH5: Having blood on toothbrush after brushing teeth	1517	70.30 (35.80)	0.48	1.41	1.27

**Table 3:** Threshold disordering tests for PedsQL Oral Health Scale

	**Average measure**	**Step measure**	**Infit MnSq**	**Outfit MnSq**
Child self-report				
0 = almost always	-2.82	–	1.22	1.28
1= often	-1.27	-1.45	1.05	1.06
2 = sometimes	-0.12	-0.73	0.97	0.93
3 = almost never	1.23	0.30	0.95	0.95
4 = never	3.11	1.88	1.00	1.00
Parent-proxy report				
0 = almost always	-2.29	–	1.19	1.15
1 = often	-1.05	-0.71^a^	0.84	0.79
2 = sometimes	-0.19	-0.78	1.01	0.96
3 = almost never	0.94	-0.08	0.96	0.90
4 = never	2.80	1.57	1.06	1.02

^a^ The response of 1 (*often*) is disordered.

**Table 4:** Test for local dependency

**No. Item**	**No. Item**	**r**	
		**Child self-report**	**Parent-proxy report**
OH1	OH2	-0.24	-0.12
	OH3	-0.15	-0.10
	OH4	-0.16	-0.29
	OH5	-0.38	-0.42^a^
OH2	OH3	-0.24	-0.26
	OH4	-0.28	-0.20
	OH5	-0.37	-0.44^a^
OH3	OH4	-0.19	-0.12
	OH5	-0.29	-0.37
OH4	OH5	-0.14	-0.07

^a^Absolute*r* > 0.4

**Table 5:** Test for differential item functioning (DIF)

**No. Item and description**	**Difficulty**		**DIF contrast** ^a^	**SE**	**P**
Test for gender	Male	Female			
OH1: Having tooth pain	0.11	0.22	-0.11	0.08	0.17
OH2: Having tooth pain when eating or drinking something hot, cold, or sweet	0.84	0.74	0.10	0.07	0.16
OH3: Having teeth that are dark in color	0.08	0.02	0.07	0.08	0.42
OH4: Having gum pain	-0.96	-1.05	0.09	0.10	0.37
OH5: Having blood on toothbrush after brushing teeth	0.08	0.07	-0.14	0.08	0.08
Test for rater	Child	Parent			
OH1: Having tooth pain	0.16	0.16	0.00	0.05	1.00
OH2: Having tooth pain when eating or drinking something hot, cold, or sweet	0.73	0.47	-0.26	0.05	<0.001
OH3: Having teeth that are dark in color	0.05	0.01	-0.04	0.06	0.45
OH4: Having gum pain	-0.96	-1.17	-0.22	0.07	0.003
OH5: Having blood on toothbrush after brushing teeth	0.02	0.49	0.47	0.05	<0.001

^a^ DIF contrasts were calculated as *logit* of Male (Child) minus *logit* of Female (Parent). For gender, a positive DIF contrast indicates that Male has a higher item score than does Female, and *vice versa*; for rater, a positive DIF contrast indicates that the item score on children’s oral-related quality of life is higher in Child reports than Parent reports, and *vice versa*.

## Discussion

To the best of our knowledge, this is the first study using Rasch analysis to examine the psychometric properties of the recently developed PedsQL Oral Health Scale. Generally speaking, PedsQL Oral Health Scale is a promising instrument for healthcare providers to capture the OHRQoL for students. Although our results found that PedsQL Oral Health Scale contained some limitations, of majority was in the parent-proxy report, the limitations were not substantial. The major weakness for parent-proxy report was the disordering response in the category of 1 (which indicates “*often*”), while other unsatisfactory properties were minor, such as the slightly high fit statistics and local dependency; slightly low person separation index. In contrast, almost all psychometric properties of student self-report were satisfactory, except for the close-to-acceptable person separation reliability and index. No DIF items were found across gender, while three DIF-trivial items were found across student self-report and parent-proxy report, which is reasonable and acceptable.

The PedsQL Oral Health Scale has been confirmed as an appropriate OHRQoL instrument in terms of the original English version,^10^ the Brazilian Portuguese version,^11^ and the Persian version.^1^ Although the previous studies^1,10,11^ applied CTT to test the psychometric properties of PedsQL Oral Health Scale, the psychometric evidences are suggested to be reexamined using IRT or Rasch models.^12,23^ Using Rasch models helps healthcare providers to understand the psychometric properties that cannot be examined using CTT. As a result, we tried using Rasch analyses to reconfirm the feasibility and validity of PedsQL Oral Health Scale, and the results suggested that all items were embedded in the same construct (i.e., OHRQoL). In addition, our results of the satisfactory item separation reliability (0.99) outperformed the internal consistency (0.68 and 0.84 in English; 0.65 and 0.59 in Brazilian Portuguese; 0.86 and 0.81 in Persian versions) from previous CTT findings.^1,10,11^ Moreover, our person separation reliability (0.63 and 0.72) was similar to or lower than the above mentioned internal consistency values. Rasch analyses estimate the reliability separately for items and respondents, and CTT does not separate the items and respondents to estimate the reliability.^12,16^ Therefore, we concluded that the items of PedsQL Oral Health Scale are reliable, while the low internal consistency in previous studies may be attributable to respondents’ characteristics, as confirmed by our person separation reliability. The cognitive ability of children and adolescents are still under development, and parents may not fully understand their children’s oral health. Hence, person separation reliability and internal consistency were found to be low in our results and previous studies.^10,11^

Although the PedsQL Oral Health Scale exhibited the expected threshold ordering and acceptable item dependence in the student self-report, a disordered category and some high item dependencies were found in the parent-proxy report. Similarly, the parent-proxy of PedsQL Generic Core Scale is found to have two disordered categories.^28^ In the study of Amin et al,^28^ they found that the responses of “*almost never*” and “*often*” were disordered, while our results only found “*often*” being disordered. Amin et al^28^ further combined the response of “*almost never*” with that of “*never*”; the response of “*almost always*” with that of “*often*”, and found a substantial improve in the Rasch models. Though collapsing two categories into one somewhat can account for the disordered issues, we tended not doing so because we were unsure that whether “*often*” should be combined with “*almost always*” or combined with “*sometimes*”. Instead, we encouraged future empirical studies applying two sets of response (i.e., one set combined the responses of “*often*” and “*almost always*”, and the other combined those of “*often*” and “*sometimes*”) to further probe this issue.

In terms of the DIF items across gender, our results are comparable to the PedsQL Generic Core Scale^28^ that there were no DIF items. In addition, we found three DIF-trivial items across student self-report and parent-proxy report. However, we considered that this is not a serious problem because of the following reason. The difference between student self-report and parent-proxy report is well documented in many QoL instruments,^29-31^ and the trend is using the student self-report as the primary measure. Parent-proxy was used for two purposes: one is to substitute the student self-report when the student is too young or too ill to answer a questionnaire^5^; the other is to understand that whether the parents underestimate or overestimate their students’QoL.^32^ Because the DIF was not substantial, we considered that the parent-proxy report of PedsQL Oral Health Scale can fulfill the two purposes mentioned above.

There are some limitations in this study. First, all participants were recruited in the same city, with culture and socioeconomics might be various in different cities of the same country, generalizing our results to the entire Iran population should be cautioned. Second, students filled out a student self-report under the supervision of a research assistant, while parents completed a parent-proxy report at home. Therefore, we could make sure that students were concentrating while answering the questionnaire. However, we did not know whether parents paid enough attention on the questionnaire. This also somewhat explains that parent-proxy report had inferior properties to student self-report. Third, the parent-proxy reports were answered by heterogeneous raters (e.g., mothers and fathers), and the perspectives on students’ OHRQoL might be differed in different raters. Finally, we did not define the social class when recruited our participants, and this may affect our results.

## Conclusion

In conclusion, PedsQL Oral Health Scale is a promising QoL instrument to help healthcare providers understand the OHRQoL for students. The student self-report demonstrated stronger properties than the parent-proxy report did, and we followed the trend to recommend using student self-report as the primary measure. In addition, future studies are warranted to further examine the disordered issue in the parent-proxy report.

## Ethical approval

Permission for this study was obtained from the Organization for Education at Qazvin involved and the study protocol was approved by the ethics committee in the Qazvin University of Medical Sciences. Each participant was informed about the aims of the study and signed a consent form before participation and their data were kept confidential. Participation in the study was voluntary and the participants were free to leave the study at any stage.

## Competing interests

All the authors declare that there is no conflict of interests.

## Authors’ contributions

CYL and AHP were responsible for designing the study, analyzing and interpreting the data, and drafting the manuscript. AHP was responsible for data collection. CYL and SK interpreted the data, and revised the manuscript. SK participated in study conception and design and critical revision. All authors have read and approved the final manuscript.

## Appendix A. Pediatric Quality of Life Inventory™ (PedsQL™) Oral Health Scale Items

**Child Self-Report Item Content**

1. I have tooth pain.

2. I have tooth pain when I eat or drink something hot, cold, or sweet.

3. I have teeth that are dark in color.

4. I have gum pain.

5. I have blood on my toothbrush after brushing my teeth.

**Parent-Proxy Report Item Content**

1. Having tooth pain.

2. Having tooth pain when eating or drinking something hot, cold, or sweet.

3. Having teeth that are dark in color.

4. Having gum pain.

5. Having blood on toothbrush after brushing teeth.

Reproduced with permission from J.W. Varni, Ph.D. Copyright © 1998. The PedsQL™ is available at http://www.pedsql.org.
